# The negative footprint illusion in environmental impact estimates: Methodological considerations

**DOI:** 10.3389/fpsyg.2022.990056

**Published:** 2022-10-03

**Authors:** Patrik Sörqvist, Mattias Holmgren

**Affiliations:** Department of Building Engineering, Energy Systems and Sustainability Science, University of Gävle, Gävle, Sweden

**Keywords:** negative footprint illusion, methodology, response format, scale, cognitive bias

## Abstract

Past research has consistently shown that carbon footprint estimates of a set of conventional and more environmentally friendly items in combination tend to be lower than estimates of the conventional items alone. This ‘negative footprint illusion’ is a benchmark for the study of how cognitive heuristics and biases underpin environmentally significant behavior. However, for this to be a useful paradigm, the findings must also be reliable and valid, and an understanding of how methodological details such as response time pressure influence the illusion is necessary. Past research has cast some doubt as to whether the illusion is obtained when responses are made on a ratio/quantitative scale and when a within-participants design is used. Moreover, in past research on the negative footprint illusion, participants have had essentially as much time as they liked to make the estimates. It is yet unknown how time pressure influences the effect. This paper reports an experiment that found the effect when participants were asked to estimate the items’ emissions in kilograms CO_2_ (a ratio scale) under high and under low time pressure, using a within-participants design. Thus, the negative footprint illusion seems to be a reliable and valid phenomenon that generalizes across methodological considerations and is not an artifact of specific details in the experimental setup.

## Introduction

Past research has consistently shown that carbon footprint estimates of a set of conventional and more environmentally friendly items in combination tend to be lower than estimates of the conventional items alone. This phenomenon has been coined the ‘negative footprint illusion’ (see Sörqvist et al., 2020, for a review). An averaging bias appears to be responsible for the illusion, whereby people average vices (e.g., conventional buildings) and virtues (e.g., “green” buildings) when they make estimates of the items in combination, rather than making a summative estimation (Holmgren et al., 2018a). This explanation is reinforced by the fact that the negative footprint illusion disappears when participants are primed to think in a summative manner (Holmgren et al., 2021). The negative footprint illusion is a benchmark for the study of how cognitive heuristics and biases underpin environmentally significant behavior. However, for this to be a useful paradigm, the findings must also be reliable and valid, and an understanding of how methodological details influence the illusion is necessary.

The negative footprint illusion appears to be quite robust to many methodological considerations. For example, it does not seem to matter much whether the estimates concern food (Gorissen and Weijters, 2016; Kusch and Fiebelkorn, 2019; but see Threadgold et al., 2022), vehicles (Kim and Schuldt, 2018), or buildings (Holmgren et al., 2018a). It has also been shown in both within-participant designs (Holmgren et al., 2018b; but see Gorissen and Weijters, 2016) and between-participant designs (Holmgren et al., 2018a). The illusion seems therefore to be robust to some variations in the experimental setup. In turn, the illusion varies in size depending on the spatial distribution of the conventional and environmentally friendly items (Sörqvist et al., 2022) and it seems to vary in size with dispositional factors (MacCutcheon et al., 2020; Threadgold et al., 2022).

The response scale is one methodological consideration that is of particular interest to the current study. Asking participants to make the responses on a 9-point scale with endpoints labeled (very low impact vs. very high impact), or on a 9-point scale in which each point is labeled with a CO_2_ value, seems to matter little (Gorissen and Weijters, 2016; see also Holmgren et al., 2018a). Requesting the participants to make “indirect” estimates of the carbon footprint, by asking them to estimate the number of trees (which binds carbon) needed to compensate for the emissions from the items results in just the same. There is a tendency to assign a smaller number of trees to a combination of environmentally certified and conventional buildings in comparison with the conventional buildings alone (Holmgren et al., 2018b). However, there is still reason to believe that the response format may influence the respondents’ behavior (Weijters et al., 2010), in particular, if the response format is ambiguous to the participant.

With one exception (Holmgren et al., 2018b), all previous studies on the negative footprint illusion (Gorissen and Weijters, 2016; Holmgren et al., 2018a, 2021; Kim and Schuldt, 2018; Kusch and Fiebelkorn, 2019; MacCutcheon et al., 2020; Threadgold et al., 2022) have asked participants to make their estimates of environmental impact or carbon footprint on an ordinal, limited response scale. That is, on a scale ranging from, for example, 1–9 in which the possible responses are quite limited, the lowest value does not represent “0 carbon footprint/emissions/kg CO_2_” and the size of the differences between the steps on the scale are ordinal rather than identical. This circumstance cast doubt as to whether the negative footprint illusion is truly a manifestation of cognitive biases or actually just a consequence of ambiguous task instructions and scales. For example, participants might activate a qualitative mindset when making responses on an ordinal/qualitative scale (*cf.* Gorissen and Weijters, 2016). When in this mindset, participants might interpret the task as if they should estimate how “good” or “bad” the item set is for the environment, rather than estimating the quantitative amount of carbon emissions. It could be argued that conventional items in combination with “green” items are indeed better for the environment than the conventional items alone, depending on perspective, and consequently, participants would be accurate in their qualitative evaluation of the items. Because of this, it is both methodologically and theoretically important to test whether the negative footprint illusion emerges when estimates are made on a quantitative/ratio scale. The current study aimed to test whether the negative footprint illusion is obtained when participants are asked to make their estimates on a ratio scale, in which “0” represents complete absence of emissions/kg CO_2_, the size of the difference between scale steps is identical, and there is essentially no reason to believe that participants have misinterpreted the response scale.

A second methodological consideration of interest to the current study is response time pressure. Cognitive biases often become stronger when decisions and judgments must be made quickly and under time pressure (Roberts and Newton, 2001; Evans St. and Curtis-Holmes, 2005; Hilbig et al., 2012; Dekel and Sagi, 2020). In previous studies on the negative footprint illusion (e.g., Gorissen and Weijters, 2016; Holmgren et al., 2018a,b), the participants have had essentially as much time as they liked at their disposal to make the estimates. It is therefore yet unknown whether the negative footprint illusion becomes larger under time pressure, although it would be useful from both a theoretical and methodological viewpoint to know whether the effect behaves as expected from past research on the effects of time pressure on cognitive biases or if it behaves differently. The current study tested the effect of time pressure on the negative footprint illusion by comparing rapid responses with slow responses because it would reveal important information about the basic mechanisms behind the effect.

Finally, a third methodological consideration of interest here was the choice of experimental design. Previous research (Gorissen and Weijters, 2016) has been somewhat doubtful as to whether the negative footprint illusion can really be found in a within-participants design, presumably because within-participants designs allow participants to remember and compare their own estimates between conditions. The current study used a within-participants design to build further evidence on this issue.

In sum, the experiment aimed to test whether the negative footprint illusion can be detected when responses are made on a ratio scale, which has never been shown before. The time that was available for the participants to make their responses was manipulated to test whether time pressure influences the magnitude of the effect. And a within-participants design was selected to test whether the negative footprint illusion—typically studied in between-participant designs—generalizes to this design choice.

## Materials and methods

### Participants

A total of 120 participants were recruited to take part in the experiment. Eighteen of them were removed prior to the analysis for reasons detailed below, resulting in a final sample of 102 participants (70% women, mean age = 34.25 years, SD = 10.94 years). The experiment was distributed by the crowd-sourcing platform Prolific academic. The inclusion criteria were to be between 18 and 65 years of age and living in the United Kingdom. All participants received a payment rate of around £8 per hour for their participation (note that participation only took a few minutes) and participated under informed consent.

### Materials

Data were collected by an online questionnaire created by the web-based survey instrument Qualtrics. After reading an information sheet (informing the participants that participation is voluntary and that they can withdraw from the study at any time) and responding to the consent form, the participants received information stating: *“In this survey you will be asked to make different kinds of estimates under a short time frame of 5 s. In the first block, you will be asked to make estimates related to colour-discrimination and in the second block you will be asked to make estimates related to CO_2_-emissions. Before each block starts you will receive more information pertaining to that block. Please take your time and read the instructions carefully before proceeding.”* Note that in the short response time window condition, the participants were told they had 5 s to respond as described above. In the long response time window condition, the participants were instead told they had 50 s to respond, all else being equal. The 5 s limit was selected because a pilot study with a handful of participants suggested that 5 s (but not less) was needed for participants to have enough time to be able to make the estimates.

#### The training block

On the next page of the questionnaire, participants were introduced to a training block, which was constructed to make the participants familiar with the response format. In the training block, the participants were presented with images with various shades of gray, white, and black. These stimuli were chosen because they were clearly different from the stimuli used in the main task (see below) to avoid potential interference between the training block and the main block, while still allowing the participants to become acquainted with the response format. The information presented to the participants read: *“On the next slides you will see images. Your task is to estimate whether the image is dominantly black or white. You will be making each estimates by first clicking on the text box, then typing in a number from 0 to 99 by using the keyboard on your device. The lower estimates indicate “dominantly white” and the higher estimates indicate “dominantly black.” For example a score of 0 would be a completely white picture whereas a score of 99 would be a completely black picture. You will have five seconds to respond to the question. Have your fingers ready to type in your response before continuing to your first estimate.”* In the long response time window condition, the participants were instead told they had 50 s to respond, all else being equal. After reading the information, the participants proceeded through 10 trials where they were asked to estimate whether a picture was dominantly white or black. Between each trial, they received a text stating: *“When you are ready to make the next estimate, click on the arrow below.”*

#### The main task

When they had completed the training block, the participants were introduced to the critical judgment task. Before starting, they received information stating: *“On the next slides you will see several houses together. You will see two types of houses: conventional (having a yellow colour) and environmentally certified (having a green colour) houses. Note that environmentally certified houses produce less CO_2_ emissions compared to conventional houses. Your task is to estimate what the environmental impact is, measured in kilograms of carbon dioxide (kg CO_2_) emissions for all the houses in the image together. Your estimate should indicate the number of kilograms of CO_2_ that the houses produce together due to, for example, ventilation, heating and energy-use. Click on the arrow below to get information on how you will make your estimate!”* To increase the possibility of avoiding non-responses, they were given a detailed instruction on how they were supposed to approach the task. This instruction read: *“You will be making each estimate by first clicking on the text box, then typing in a number from 0 to 99 by using the keyboard on your device. Remember, the number you type in in the text box should indicate the number of kilograms of CO_2_ emissions the houses produce. A higher number is worse for the environment compared to a low number. You will have five seconds to respond to each question. Before moving on, have your fingers ready to type in your response before continuing to your first estimate!.”* After reading this, they were introduced to seven trials consisting of pictures depicting either only conventional buildings or conventional buildings together with “green” buildings. They made their estimates by typing in the estimates, ranging from 0 (kilograms of carbon dioxide emissions) to 99 (kilograms of carbon dioxide emissions), in a text box. A digital clock counting down from 5 s (or 50 s, depending on condition)was shown during each trail. If the time expired before the participants were able to make an estimate, the computer continued automatically to the next trial. The first trial was not included in the analysis as it was used to make the participants used to the, slightly different, response format and stimuli. Between each trial, they received a text stating: *“When you are ready to make the next estimate, click on the arrow below.”*

### Design and procedure

A mixed within-between participants design was used with two independent variables: display of buildings with two levels (only conventional buildings [conventional only condition] vs. conventional + “green” buildings [“green” addition condition]) and response time window with two levels (5 s vs. 50 s). The order between the two display conditions was counterbalanced between participants. More specifically, the participants were randomly assigned to either starting with a trial consisting of only conventional buildings or a trial consisting of conventional + “green” buildings.

Moreover, three trials consisted of conventional buildings together with “green” buildings (75 conventional buildings + 25 green buildings; 20 conventional buildings + 20 “green” buildings; 15 conventional buildings + 5 “green” buildings) and three trials consisted of only conventional buildings (75 conventional buildings; 20 conventional buildings; 15 conventional buildings). In the analyses, an average for each participant was calculated for the responses in the display condition with items of both types, to obtain a single measure of kg CO_2_ estimates in that condition for each participant, respectively. A similar calculation was made for the response in the display condition with only conventional items. Seventeen of the participants in the “5 s response time window” condition failed to make all six responses and they were therefore removed prior to the analysis, resulting in a final sample in that condition of 43 participants. One of the participants in the “50 s response time window” condition failed to make all six responses and was therefore also removed prior to the analysis, resulting in a final sample in that condition of 59 participants.

## Results

**Figure 1:**
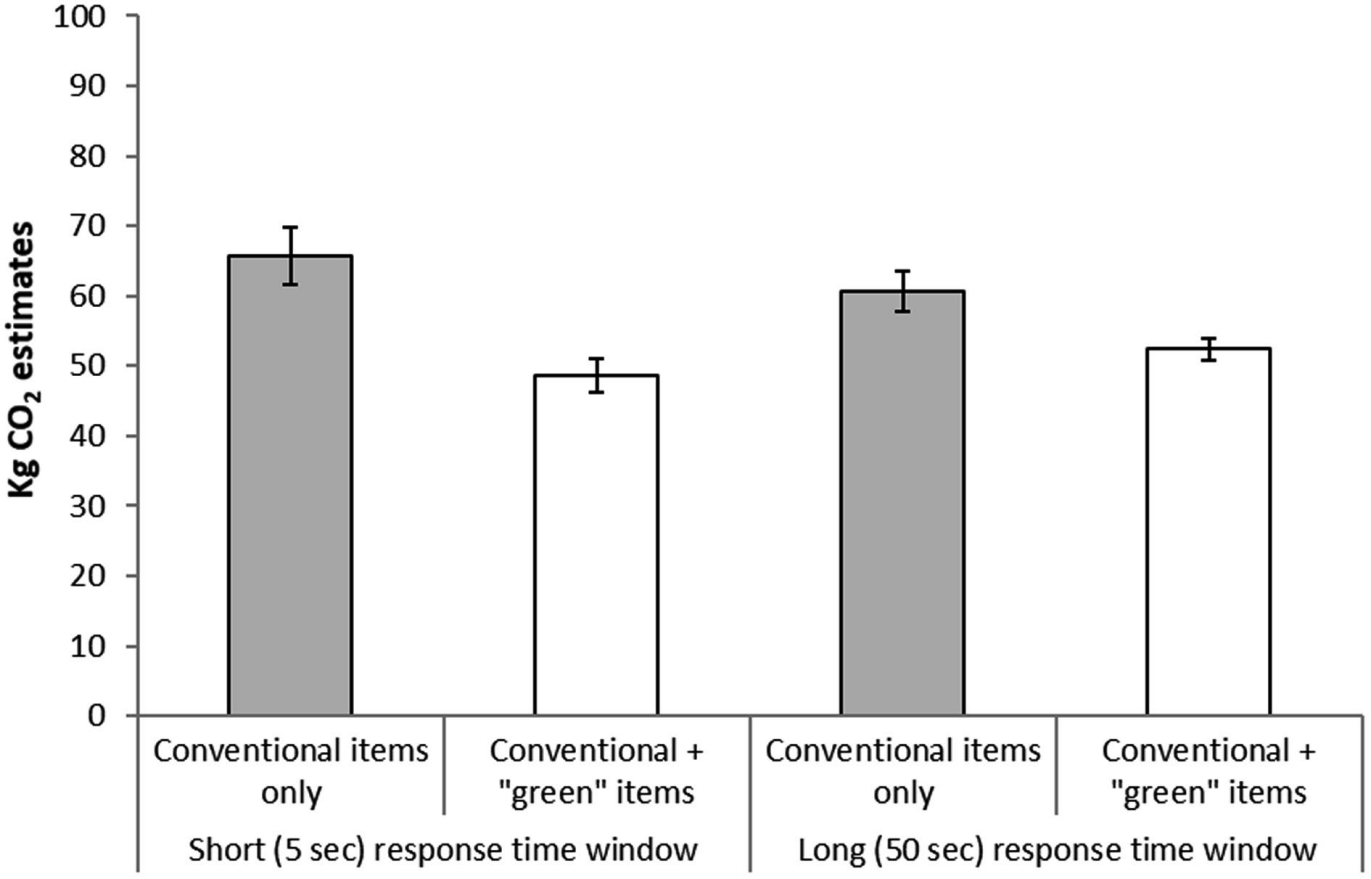
The figure shows mean estimates of kg CO_2_ of sets of items either comprising conventional items only or comprising conventional items and “green” items in combination. Estimates were either made under high time pressure (5 s) or under low time pressure (50 s). Error bars represent standard error of means.

As shown in Figure 1, the typical negative footprint illusion was found in both response time conditions. Moreover, the effect was slightly larger in the short (5 s) response time window condition in comparison with the long (50 s) condition. The participants in the “5 s response time window” condition assigned more CO_2_ to the conventional only items (*M* = 65.78 kg CO_2_, SD = 26.72) in comparison with how much they assigned to the conventional items in combination with “green” items (*M* = 48.66 kg CO_2_, SD = 15.41). This difference between conditions was statistically significant, *t* (42) = 4.63, *p* < 0.001. Similarly, the participants in the “50 s response time window” condition assigned more CO_2_ to the conventional only items (*M* = 60.59 kg CO_2_, SD = 22.29) in comparison with how much they assigned to the conventional items in combination with “green” items (*M* = 52.41 kg CO_2_, SD = 12.29). This difference between conditions was also statistically significant, *t* (58) = 4.06, *p* < 0.001. A 2(display of buildings) × 2(response time window) analysis of variance with CO_2_ estimates as dependent variable indicated that the difference between the two display conditions was larger in the “5 s response time window” condition, in comparison with the size of the difference in the “50 s response time window” condition as the interaction between the two factors was significant, *F* (1, 100) = 5.15, *p* = 0.025. However, this interaction has to be treated with caution. Ten participants in the “5 s response time window” condition and seven participants in the “50 s response time window” condition made estimates of 99 kg CO_2_ (the maximum estimate) in the conventional only display condition, suggesting that the interaction could potentially reflect a ceiling effect. When these participants were removed, the participants in the “5 s response time window” condition still assigned more CO_2_ to the conventional only items (*M* = 55.72 kg CO_2_, SD = 22.09) in comparison with how much they assigned to the conventional items in combination with “green” items (*M* = 46.72 kg CO_2_, SD = 14.99); a difference that was still statistically significant, *t* (32) = 2.56, *p* = 0.016. Similarly, the participants in the “50 s response time window” condition assigned more CO_2_ to the conventional only items (*M* = 55.43 kg CO_2_, SD = 18.31) in comparison with how much they assigned to the conventional items in combination with “green” items (*M* = 50.38 kg CO_2_, SD = 11.64), *t* (51) = 2.66, *p* = 0.010. However, an analysis of variance indicated that the interaction between the factors was not still significant, *F* (1, 83) = 1.15, *p* = 0.286.

## Discussion

The first conclusion that can be made from the experiment reported here is that the negative footprint illusion can be detected when participants make their estimates on a ratio scale. While response format choices of past studies on the negative footprint illusion may cast some doubt on how the participants interpreted the task (*cf.* Gorissen and Weijters, 2016), the results reported here, together with the plethora of studies on this illusion published until now (Sörqvist et al., 2020), suggest that the illusion is quite robust to such details. If the negative footprint illusion had not been found with the response format used in the current study, it had been possible to argue that the effect found with ordinal response scales (e.g., Gorissen and Weijters, 2016; Holmgren et al., 2018a) is an artifact of the response scale—perhaps an ordinal response scale promotes a qualitative mindset while a ratio response scale promotes a quantitative mindset, or perhaps the participants do not fully understand the task. Finding the negative footprint illusion with a ratio response scale as in the current experiment suggests that the effect is rather a consequence of a cognitive bias (presumably an averaging bias) in environmental impact estimates, not an artifact of methodological peculiarities.

The experiment reported here also shows that the illusion is robust to a number of other methodological considerations. The illusion has mostly been studied in the context of between-participants designs with a few exceptions (Holmgren et al., 2018b; Threadgold et al., 2022) and one study in the past (Gorissen and Weijters, 2016) failed to find the effect in a within-participants design, while it was obtained in a between-participants design. The results reported here suggest that the illusion is robust also to this methodological choice. We can only speculate on the reason why the effect was found in the experiment reported here and not in the experiment by Gorissen and Weijters (2016). One possibility is that the fractional factorial design used by Gorissen and Weijters (2016), wherein each participant estimated a subset of a total of 24 stimulus sets, introduced too much error variance. In the current study, all participants made estimates of the same 6 stimulus sets, perhaps introducing less error variance.

Finally, while past research has allowed the participants unlimited time to make their estimates, the current study shows that the illusion is also found when participants are required to make hasty responses. If anything, the illusion seems to be larger when hasty responses are required, in line with previous research suggesting that cognitive biases become exacerbated under time pressure (Roberts and Newton, 2001; Evans St. and Curtis-Holmes, 2005; Hilbig et al., 2012; Dekel and Sagi, 2020). Under time pressure, estimates arguably rely more heavily on intuitive thinking. Participants do not have time to carefully think it through and realize that a set of conventional items must cause fewer kg CO_2_ than the very same set of conventional items plus another set of “green” items. Instead, they become more susceptible to the averaging bias.

### Limitations

One limitation of the current study is that these estimates were made under uncertainty. The negative footprint illusion may well be constrained to situations where people are asked to make estimates about something they do not have enough knowledge about to make accurate estimates. In the case reported here, participants were asked to estimate the amount of CO_2_ that is generated by a number of houses. The general population (presumably) do not know the actual answer to this question. Future research could investigate if the illusion disappears with a higher level of certainty, by, for example, teaching participants about how much CO_2_-emissions an average house produces. Participants may also be less susceptible to the negative footprint illusion when to-be-estimated items come from different categories, in particular, if the items belong to a category that participants are more knowledgeable about. There is empirical evidence that supports this idea. For example, Threadgold et al. (2022) found a negative footprint illusion in estimates of buildings and in estimates of cars but not in estimates of apples. It should be noted though, that the negative footprint illusion has been found in a sample comprising of experts (Holmgren et al., 2018b). This indicates that a higher level of knowledge in the judgmental domain does not necessarily make one immune to the effect, at least not when the estimates are made on intuitive rather than reflective thinking.

Another limitation that should be addressed is the loss of 18 participants from the full sample of 120 participants, due to a relatively high rate of participants not being fast enough to make all estimates within the given time window. A reason for this could be that the participants lacked proper task-related knowledge needed to make hasty responses. Regardless of the reason, the drop rate could have compromised the data in unpredictable ways. A further complication was that 17 participants reported 99 kg CO_2_ as their estimates of all conventional only stimulus sets. However, with these participants removed from the analysis, the negative footprint illusion was still present in both response time window conditions. This is important since it shows that three of the main findings from the current study were not compromised by this issue: the fact that the negative footprint illusion is found with a ratio response scale, in a within-participants design and when estimates are made under high time pressure. Whether the negative footprint illusion is larger under high time pressure is less clear though.

### Conclusion

The negative footprint illusion is not a consequence of participants misinterpreting the response scale. The paradigm can be used as a reliable benchmark for the study of cognitive heuristics and biases underpinning environmentally significant behavior.

## Data availability statement

The raw data supporting the conclusions of this article will be made available by the authors, without undue reservation.

## Ethics statement

Ethical review and approval was not required for the study on human participants in accordance with the local legislation and institutional requirements. The patients/participants provided their written informed consent to participate in this study.

## Author contributions

PS and MH designed the experiment. MH conducted the data collection and analysis of the data and wrote parts of the manuscript. PS wrote large parts of the manuscript. All authors contributed to the article and approved the submitted version.

## Conflict of interest

The authors declare that the research was conducted in the absence of any commercial or financial relationships that could be construed as a potential conflict of interest.

## Publisher’s note

All claims expressed in this article are solely those of the authors and do not necessarily represent those of their affiliated organizations, or those of the publisher, the editors and the reviewers. Any product that may be evaluated in this article, or claim that may be made by its manufacturer, is not guaranteed or endorsed by the publisher.
